# A survey of wild plant species for food use in Sicily (Italy) – results of a 3-year study in four Regional Parks

**DOI:** 10.1186/s13002-015-0074-7

**Published:** 2016-02-09

**Authors:** Mario Licata, Teresa Tuttolomondo, Claudio Leto, Giuseppe Virga, Giuseppe Bonsangue, Ignazio Cammalleri, Maria Cristina Gennaro, Salvatore La Bella

**Affiliations:** Department of Agricultural and Forest Sciences, Università degli Studi di Palermo, Viale delle Scienze 13, 90128 Palermo, Italy

**Keywords:** Wild plant species, Food use, Sicilian Regional Parks, Traditional plant knowledge, Cultural importance index

## Abstract

**Background:**

This paper illustrates the results of a study carried out in four Regional Parks of Sicily (Italy), concerning traditional knowledge on food use of wild plant species. The main aims of the paper were: (i) to verify which wild plant species are used for food purpose in the local culture based on information provided by elderly inhabitants (ii) to verify the presence of wild plant species which have not been cited for food use in previous studies in the Mediterranean area (iii) to determine how many of the most frequently cited wild plant species are cultivated by the local population in the four Sicilian Parks.

**Methods:**

Semi-structured interviews were carried out in the local communities of the four Regional Parks between 2007 and 2010. A total of 802 people over the age of 60 were interviewed. Cultural Importance Index was used to evaluate the level of importance given to any wild plant species as a food in the local culture. The level of appreciation of the wild plant species and the possible effects of wild plants on human health were also investigated.

**Results:**

Local communities currently use a total number of 119 wild species for food purposes. Asteraceae and Brassicaceae were the most represented botanical families. In each of the four Sicilian Parks, *Cichorium intybus* L. and *Foeniculum vulgare* Mill. obtained the highest Cultural Importance Index values. Sixty-four species were indicated as also having medicinal properties. Leaves and other aerial plant parts were the parts most-used for the preparation of traditional recipes.

**Conclusions:**

The research shows that the level of traditional knowledge on the food uses of wild plant species in the study area is poor. The food uses of plants which are most likely to survive over time are those at the interface of food and medicine. Further agronomic studies are needed for a number of species with a view to introducing them as a crop into non-intensive agricultural systems.

## Background

Wild plant species have always played a fundamental role in the diet of man. Although plants once represented a good source of food in rural areas, there has been a gradual change in lifestyle over the centuries; people have moved away from the countryside and there has been huge progress in farming methods. Wild plants became a progressively less important source of food over time, as it was replaced by food products from intensive farming crops and industrialized processing. Food habits and tastes have undergone intense change, with the introduction of increasingly more refined and highly-processed industrial food products. In more recent times, the consumption of food products of animal origin has increased sharply, giving rise to a series of health problems, exacerbated by a fall in the consumption of plant foods rich in fiber, vitamins and trace elements [1, 2]. Furthermore, a substantial increase in the use of pesticides and fertilizers is threatening the ecosystem, upsetting plant biodiversity and causing a fall in wild plant species numbers [3–7]. In an effort to highlight the importance of wild plant species in our diets, a number of studies have been carried out in recent years in the Mediterranean area documenting the nutritional and medicinal properties of these plants [8–16]. Compared to cultivated a number of wild plant species have been reported to contain greater levels of fiber [13], to have far greater antioxidant and flavonoid levels [17–19] and to contain a smaller amount of lipids [20]. A number of studies maintain that the carbohydrate, fibre, polyphenol, protein, mineral, vitamin and ω-3 fatty acid content [21–30] of various parts of the wild plants can have beneficial effects on human health. This reinforces the concept of food as medicinal, first expressed by Hypocrites in 400 BC [28]. The well-documented health properties of wild food plants have also contributed to increasing their importance as a part of the Mediterranean diet [31, 32]. This nutritional model, based on the consumption of cereals, legumes, vegetables, fresh fruit and olive oil, is recognized on a nutraceutical level throughout the world and has also been designated in recent years as a UNESCO Masterpiece of the Oral and Intangible Heritage of Humanity (2010). It seems clear from the previously cited ethnobotanical studies that a large number of wild plant species are consumed as food in various different areas of the Mediterranean: proof of the existence of knowledge and traditions linked to autochthonous ecological and cultural factors [33], and of the role that wild plant species have had in the various cultures and ethnic groups [13, 34]. In Sicily (Italy) – an island with a high level of plant biodiversity - much has been written on the food/medicinal use of wild plant species [2, 35–47]. Lentini and Venza [14] gave data and information on 188 wild plant species used in traditional Sicilian cuisine. However, quantitative analysis of the data does not appear in literature and the cultural importance of the food use of wild plants in Sicily had not been determined previous to this study. The discovery of rare wild plant species and the estimation of their cultural significance constitute an innovative aspect of the research in this field. This paper reports the results of a study on the food use of wild plant species in 4 Regional Parks in Sicily. The study includes only shrub and herbaceous species, collected from various natural areas in the 4 Parks. The areas encompassed in the study were the Madonie Regional Park, Nebrodi Regional Park, Etna Regional Park and the Monti Sicani Regional Park. The main aims of the paper were: (i) to verify those wild plant species used for food purpose by the local culture within 4 Sicilian Parks (ii) to identify any wild plants not mentioned in previous studies in the Mediterranean area as regards their food use (iii) to determine how many wild food plants are/could be cultivated locally in each of the 4 Sicilian Parks.

## Methods

### Research area

**Fig. 1:**
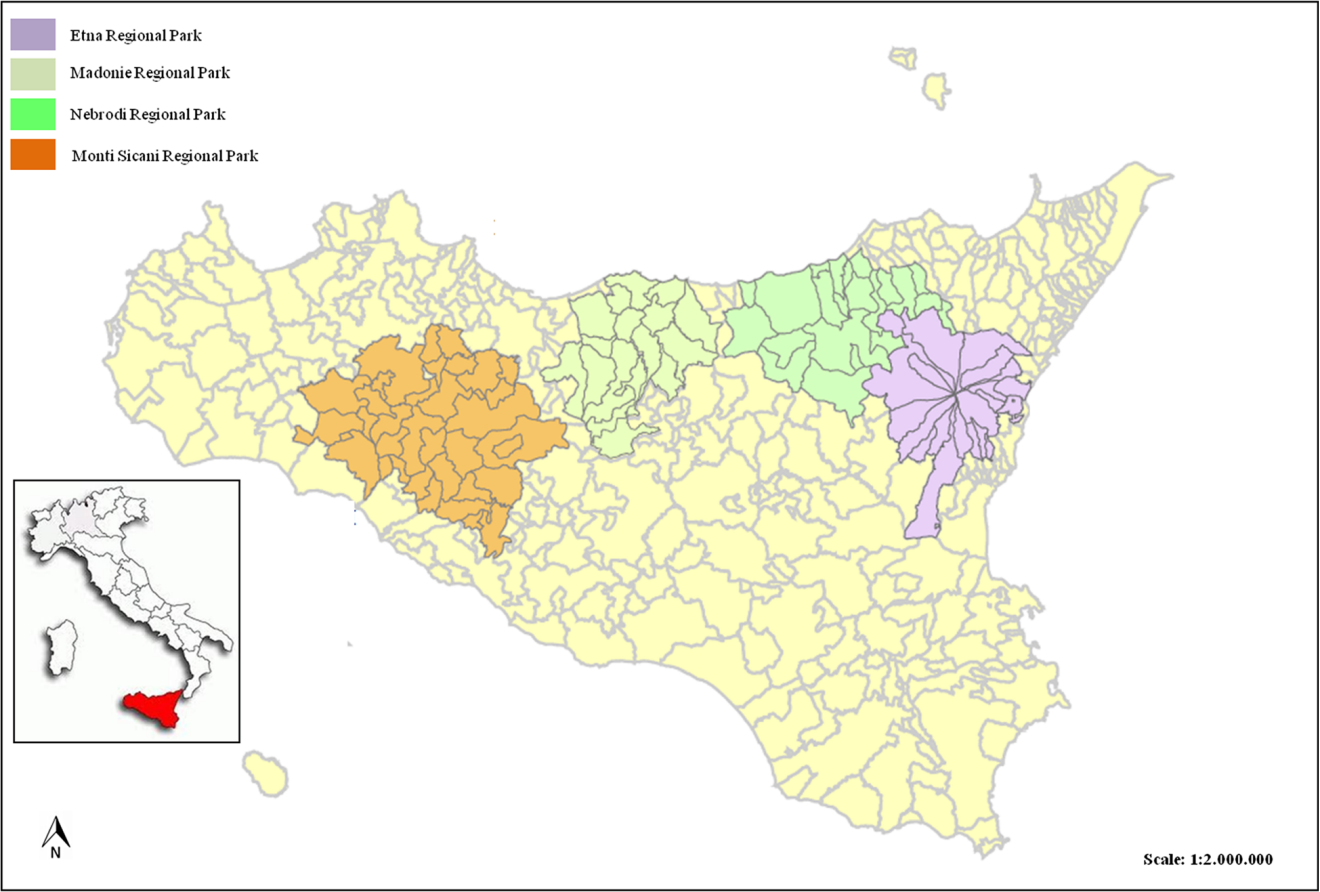
Map of the study area, showing Etna, Madonie, Nebrodi and Monti Sicani Regional Parks

The study area covered four mountainous, hinterland areas in Sicily (Italy): the Madonie Regional Park (Central Sicily), Nebrodi Regional Park (North-Eastern Sicily), Etna Regional Park (Eastern Sicily) and Sicani Regional Park (Central-Western Sicily) (Fig. 1). Based on the Rivas-Martinez bioclimatic index [48], the study area ranged from an upper thermo-Mediterranean, lower-subhumid coastal environment to an upper supra-Mediterranean, upper-humid at the higher altitudes, bioclimatic zones.

The Madonie Regional Park (37°45’ 38°03’ N latitude, and 13°50’ 14°12’ E longitude) encompasses 15 towns (altitudes ranging from 0 to 1979 m a.s.l.) and extends over an area of 400 km^2^ [49, 50]. There are 170 endemic plant species in the area [51], accounting for approx. 50 % of all Sicilian endemic species. A substantial part of the park is woodland (25 %) including typically Mediterranean species such as *Quercus ilex* L. and *Quercus suber* L., and central and northern European species, such as *Ilex aquifolium* L.*, Fagus sylvatica* L. and *Quercus petraea* (Matt.) Liebl. Crops are grown on relatively small plots and include olives, grapes, pome fruits, stone fruits and vegetables.

The Nebrodi Regional Park (37°56’07.2” N latitude, 14°42’19.2” E longitude) covers an area of 856 km^2^ and is the most extensive protected natural area in Sicily, encompassing 23 towns. Park altitudes vary considerably: the lowest areas are only a few metres above sea level but the Park is also home to Monte Soro, which soars 1.847 m above sea level [52]. Although the Park covers less than a tenth of the Island (8 %), examples of nearly half of all island species can be found here (45 %); many of which of considerable taxonomic and phyto-geographical interest. A particularly high number of regional endemisms can be found amongst the Nebrodi mountain flora, such as the Nebrodi broom (*Genista aristata* C. Presl.), the Nebrodi Carline Thistle (*Carlina nebrodensis* Guss. ex DC.), the Boccone Hellebores (*Helleborus bocconei* Ten.) and the Boccone Turkey Oak [*Quercus gussonei* (Borzí) Brullo]. Cereal and fodder crops are grown in the area, together with a substantial number of olive, fruit and horticultural crops. The animal farming sector is also fairly consistent, a factor which affords environmental and economic protection.

Etna Regional Park was the first national Park to be established by the Region of Sicily. It is 590 km^2^ and encloses 20 towns. The Park’s most important resident is without doubt Mount Etna, the highest and most active volcano in Europe, declared a World Heritage Site by UNESCO in June 2013. The land in the park is highly varied. There are large areas of fertile soils with dense woodland or shrubby, meadow areas, and, in stark contrast, vast areas where fresh lava gathers and where no vegetation grows. The most abundant forest species, found at most altitudes on Etna, are *Quercus ilex*, and the deciduous oaks belonging to the *Quercus robur* L. group : *Quercus cerris* L., *Castanea sativa* Miller, *Fagus sylvatica* L., *Betula pendula* Roth and *Pinus nigra* subsp. *laricio* Maire [53]. Agriculture in the Etna area is known for its high quality and large variety of fruit crops, thanks to a number of ancient varieties which have survived over the centuries. Most common crops are apple, citrus, chestnut, pistachio, vine and olives.

The Monti Sicani Regional Park (37.40-37.87 latitude; 13.18-13.63 longitude) covers a surface area of about 230 km^2^ and includes 32 small towns [54]. The altitudinal range of this study area is from 300 to 1613 m a.s.l. A total of 850 vascular plants grow in the Park, 68 (9.5 %) of which are endemic to the park. The woodlands areas are vast, mainly populated by evergreen and deciduous oaks (*Quercus ilex, Quercus suber*, *Q*uercus *gussonei* and *Quercus pubescens* Willd. s.l.) Forests border on traditional olive groves, fruit orchards and crop fields growing cereals, fodder, and vegetables. are also common.

### Field interview methods

The research was carried out by conducting interviews in the 90 towns of the 4 Sicilian Parks. The interviewers selected elderly people who had spent their entire lives in the area and who were/had been traditional farmers (agriculture and livestock farming). The interview protocol is described in [36–39]. Interviews followed a semi-structured format [55]. The study was carried out following the ISE code of Ethics [56], informants were made aware of the scope of the study and Prior Informed Consent [57] was requested verbally. The conversations took place in Italian. However, in some cases, Sicilian dialect was used. The interviewees were asked to speak freely about wild food species in order to acquire a list of those species used. The following questions were asked during the interview: *Which plants have you used in your lifetime and which do you continue to use today for food purposes*? *Which of these plants used for food purposes have you also used for other reasons? How do you use the plants? Where do you gather these plants? How useful do you consider these plants to be for food purposes? Do you know the cultivation techniques required for these plants Do you use any wild food plants also for medicinal purposes, etc.* The number of wild plant species which are/could be cultivated by the local populations, the level of appreciation of wild plants compared to cultivated plants and the possible effects on human health were also investigated. The plants collected were used to prepare exsiccata in the laboratories of the Department of Agricultural and Forest Sciences at the University of Palermo. The plants were identified using the Italian Flora [58]. Plant nomenclature was verified by consulting online databases, such as theplantlist.org [59] and tropicos.org [60]. Voucher specimen codes were created and specimens deposited at the Corissia Research Centre Herbarium of Sicilian Regional. This study was part of the regional project “Environmental and plant resources in the Mediterranean: study, valorisation and defence”, which took place from 2007-2011, funded by the Sicilian Regional Ministry of Agriculture and Food Resources (Italy). The main aims of the project were to select wild plant species which are of agricultural interest from Sicilian flora, and to improve knowledge on food, medicinal and handicraft uses of the species based on information collected from the local culture.

### Analysis of the data

In order to verify the existence or not of previous citations of a given plant species for food use in the Mediterranean area, and to compare food uses with other studies, literature from other regions in Italy and other Mediterranean countries was consulted. From the information gained, we were able to determine a series of data, such as the most cited species, the most cited food uses, the most used parts of the plants and the most common culinary uses. By applying the Cultural Importance Index (C.I.) [15], we were then able to estimate the cultural significance of each species, that is to verify, in quantitative terms, to what extent each species is present in the local culture and in the memory of the inhabitants. This index was calculated using the following formula:

1 $$ {\displaystyle \sum_{i=1}^{i= NU}\frac{URi}{N}} $$

it was obtained by summing the use reports (UR) in every use-category (*i* varies from only one use to the total number of uses, NU) obtained for any given species, divided by the number of informants in the interviews (N).

## Results and discussion

### Informants

A total of 658 men (82 %) and 144 women (18 %) were interviewed. The informants were aged between 60 and 90, giving and the average informant age was 73.9 years. The informants aged 70-80 years were able to supply the greatest information on the plants in terms of numbers of plants cited; less information was obtained by the younger and older age brackets. Regarding the lack of information provided by the older informants, this was in large part due to communication difficulties which arose during interviews. A far greater number of men than women were interviewed in the 4 Sicilian Parks and, therefore, the female contribution to the acquisition of information was low. This male prevalence was not intentional and was mainly due to the fact that the elderly men were more available/willing to participate. The significant lack of women in the survey we did not consider to be of decisive importance regarding information on the number of wild food plants as the men had spent most of their lives in the countryside and forests and had expert knowledge of the plants. The under-representation of women, however, may have contributed to a smaller amount of detailed information on the processing and cooking methods of wild food plants. Confirmation of this may come from Hardy’s theory [61] which maintains that the dissemination of traditional knowledge is prevalently female. All the informants said they had been resident in the area of study since birth. 72 % of informants were either retired or working farmers, foresters or herdsmen and 28 % were craftsmen, shopkeepers, teachers and housewives.

### General data on wild food plant species

A total of 119 wild shrub and herbaceous plant species were reported as being used for food purposes (Appendix). The species belong to 34 families. For each of the species the scientific name, voucher specimen code and folk names are listed. Ethno-biological information is provided by the plant parts used, preparation methods, the food/medicinal use of species, the number of citations, the presence of the species in the Sicilian Parks and the level of perceived usefulness. Similarities in the *use* of wild food species between the investigated area and those reported in studies in Sicily, in other Italian regions and some Mediterranean countries are also shown. The most represented families are Asteraceae (33 species), Brassicaceae (14 species), Lamiaceae (10 species), Asparagaceae (8 species) and Leguminosae (6 species). Only 4 species with food properties from the study were identified as being endemic to Sicily, according to [51]: *Asperula rupestris* Tineo, *Brassica rupestris* Raf., *Carlina sicula* Ten. and *Urtica rupestris* Guss. The predominance of Asteraceae in food use and food/medicinal use confirmed results from a number of studies carried out in Mediterranean countries [7, 11, 14] and [62–70]. In most cases, the species had similar names in dialect throughout the 90 towns included in the area of study. The informants used 187 dialectal names to indicate the 119 species in the study. The majority of the species were collected in the wild, or more rarely, gathered from the wild and then planted in kitchen gardens. Natural habitats such as roadsides, rocky slopes, dry meadows and uncultivated areas were the favourite habitats for gathering wild food species. Although cultivating the plants in kitchen gardens would potentially ensure their availability throughout the year, informants still preferred to gather the plants from the wild. Some aromatic species some, such as *Mentha* spp., are collected from natural habitats (wetlands, wet meadows), while others, such as *Rosmarinus officinalis* L. are often cultivated for food and/or medicinal purposes (Table 1).

**Table 1:** Gathering season, habitat and Sicilian endemism of 119 wild plant species from four Sicilian Regional Parks

Plant species	Gathering period	Habitat
*Allium ampeloprasum* L.	spring, winter	cultivated areas
*Allium subhirsutum* L.	spring, winter	cultivated areas
*Ammi majus* L.	spring	fields, pastures
*Anagyris foetida* L.	spring	fields, maquis, woods
*Anthriscus nemorosa* (M. Bieb.) Spreng.	spring	fields, woods
*Apium nodiflorum* (L.) Lag.	spring, summer	wetlands, wet meadows, grassland
*Arabis hirsuta* (L.) Scop.	autumn, spring, winter	pastures, roadsides, rocky slopes, walls
*Aristolochia sempervirens* L.	spring, winter	garrigue, maquis, woods
*Artemisia alba* Turra	spring, summer	dry meadows, rocky slopes
*Asparagus albus* L.	spring, winter	garrigue, uncultivates areas
*Asparagus acutifolius* L.	spring, winter	garrigue, uncultivated areas, woods
*Asparagus aphillus* L.	spring, winter	garrigue, maquis, uncultivated areas
*Asparagus horridus* L.	spring, winter	garrigue, maquis
*Asphodeline lutea* (L.) Rchb.	spring	dry meadows, pastures
*Asphodelus ramosus* L.	spring, winter	dry meadows, rocky slopes, uncultivated areas
^a^ *Asperula rupestris* Tineo	spring	rocky slopes
*Barbarea vulgaris* R. Br.	spring, summer	rocky slopes, roadsides
*Borago officinalis* L.	autumn, winter	cultivated areas
*Brassica fruticulosa* Cirillo	autumn	fields, maquis, meadows
^a^ *Brassica rupestris* Raf.	autumn, spring, summer, winter	fields, rocky slopes
*Bunias erucago* L.	autumn, spring, winter	meadows, walls
*Calendula arvensis* (Vaill.) L.	spring, winter	meadows, roadsides
*Calystegia sepium* (L.) R. Br.	spring	fields, meadows, wetlands
*Capparis spinosa* L.	spring, summer	rocky slopes, walls
*Carduus argyroa* Biv.	winter	pastures, roadsides, uncultivated areas
*Carduus corymbosus* Ten.	summer	pastures, roadsides, uncultivated areas, walls
*Carlina corymbosa* L.	spring	grassland, roadsides, rocky slopes
*Carlina gumnifera* L. (Less.)	spring, summer	dry grassland, garrigue, roadsides, uncultivated areas
^a^ *Carlina sicula* Ten.	spring	garrigue, pastures
*Carthamus pinnatus* Desf.	spring, winter	garrigue, pastures, uncultivated areas
*Centaurea solstitialis* L. subsp. schouwii (DC.) Gugler	spring, winter	roadsides, uncultivated areas
*Chondrilla juncea* L.	spring, summer	dry meadows, uncultivated areas, walls
*Cichorium intybus* L.	spring	cultivated areas
*Clematis cirrhosa* L.	spring	maquis, walls
*Clematis vitalba* L.	spring	maquis, hedges, walls, woods
*Clinopodium nepeta* (L.) Kuntze	spring, summer	meadows, uncultivated areas
*Crepis vesicaria* L.	spring, winter	roadsides, uncultivated areas, walls, wetlands
*Crocus biflorus* Mill.	autumn, spring, summer, winter	pastures, woods
*Dioscorea communis* L. Caddick. & Wikin	spring, summer	hedges, shrubby areas, woods
*Diplotaxis erucoides* (L.) Dc.	autumn, winter	cultivated areas, roadsides
*Diplotaxis harra* (Forssk.) Boiss.	summer	cultivated areas, fields
*Echium vulgare* L.	spring	pastures, roadsides
*Elymus repens* (L.) Gould.	autumn, spring, summer, winter	escarpments, meadows, uncultivated areas
*Equisetum arvense* L.	spring, winter	meadows, roadsides, shrubby areas, woods
*Erodium moschatum* (L.) L'Hér	spring	fields, rocky slopes
*Fedia cornucopiae* (L.) Gaertn.	spring, winter	cultivated areas, pastures, roadsides
*Foeniculum vulgare* Miller	spring, winter	cultivated areas, fields, meadows
*Fragaria vesca* L.	spring, summer	escarpments, woods
*Gladiolus italicus* Mill.	autumn, spring, winter	cultivated areas, rocky slopes
*Globularia alypum* L.	spring, winter	rocky slopes, woods
*Glycirrhiza glabra* L.	spring, summer	fields, maquis, woods
*Helianthus tuberosus* L.	winter	meadows, roadsides, wetlands, woods
*Heliotropium europaeum* L.	autumn, spring, winter	grassland, uncultivated area
*Helminthotheca echioides* (L.) Holub	spring	cultivated areas, roadsides
*Hyoseris radiata* L.	spring, winter	fields, pastures, roadsides
*Hypochoeris radicata* L.	autumn, spring, winter	dry meadows, pastures, roadsides
*Isatis tinctoria* L.	spring	roadsides, uncultivated areas
*Lactuca viminea* (L.) J. & C. Presl	autumn, spring, winter	hedges, roadsides, shrubby areas, uncultivated areas
*Lathyrus clymenum* L.	spring	fields, roadsides, woods
*Lathyrus sativus* L.	spring	fields, roadsides, woods
*Laurus nobilis* L.	autumn, summer	maquis, meadows, woods
*Leopoldia comosa *(L.) Parl.	spring	cultivated areas, grassland
*Lycium europaeum* L.	spring	maquis, coastal zones
*Malva sylvestris* L.	autumn, spring, winter	meadows, uncultivated areas
*Marrubium vulgare* L.	spring, summer	cultivated areas, pastures, rangeland
*Mentha pulegium* L.	spring, summer	wetlands, wet meadows
*Mentha spicata* L.	spring, summer	wetlands
*Muscari botryoides* (L.) Mill.	spring	fields, roadsides, broadleaf woods
*Nasturtium officinale* R. Br.	spring	wetlands
*Nepeta cataria* L.	spring, winter	hedges, roadsides, shrubby areas, uncultivated areas
*Notobasis syriaca* (L.) Cass.	spring, winter	pastures, roadsides, uncultivated areas
*Oenanthe pimpinelloides* L.	spring, summer	damp and dry grassland
*Onopordum illyricum* L.	autumn, spring	roadsides, uncultivated areas
*Origanum vulgare* L.	summer	escarpments, fields, hedges, maquis, woods
*Opuntia ficus-indica* (L.) Miller	autumn, spring, summer	cultivated areas, rocky fields
*Orobanche crenata* Forssk	spring	cultivated areas
*Oxalis pes-caprae* L.	spring, winter	fields, grassland, landscaped areas
*Parietaria officinalis* L.	autumn, spring, summer	roadsides, uncultivated areas, walls
*Phlomis fruticosa* L.	spring, summer, autumn	hedges, maquis, woods
*Plantago coronopus* L.	spring	escarpments, rocky slopes
*Plantago lagopus* L.	autumn, winter	escarpments, roadsides, uncultivated areas
*Polygonatum multiflorum* (L.) All.	spring	woods
*Polypodium vulgare* L.	autumn, spring, summer, winter	rocky slopes, uncultivated areas, woods
*Portulaca oleracea* L.	spring, summer	cultivated areas, meadows
*Raphanus raphanistrum* L.	autumn, spring, winter	cultivated areas, grassland, fields, roadsides
*Reichardia picroides* (L.) Roth	autumn, spring, winter	cultivated areas, dry meadows, walls
*Rosa canina* L.	autumn, spring, summer, winter	hedges, woods
*Rosmarinus officinalis* L.	autumn, spring, summer, winter	cultivated areas, escarpments, fields, hedges, maquis, woods
*Rubus ulmifolius* Schott	spring, summer	maquis, hedges, woods
*Ruscus aculeatus* L.	autumn, spring, winter	maquis, woods
*Scabiosa columbaria* L.	winter	grassland, pastures, rocky slopes
*Scolymus grandiflorus* Desf.	spring	roadsides, uncultivated areas, walls
*Scolymus hispanicus* L.	spring	roadsides, walls
*Scolymus maculatus* L.	spring	fields, roadsides, rocky slopes
*Scorzonera hispanica* L.	spring, winter	fields, rocky slopes, uncultivated areas
*Silene vulgaris* (Moench) Garcke	spring	grassland, hedges, shrubby areas, woods
*Silybum marianum* (L.) Gaertner	spring	roadsides, uncultivated areas
*Sinapis alba* L.	spring	cultivated areas, fields, roadsides
*Sinapis arvensis* L.	autumn, spring, winter	cultivated areas, fields
*Sinapis pubescens* L.	autumn, spring, winter	rocky slopes, uncultivated areas
*Sisymbrium rio* L.	spring, winter	fields, roadsides
*Smilax aspera* L.	spring	fields, hedge, maquis, walls
*Sonchus asper *(L.) Hill	spring, winter	roadsides, uncultivated areas
*Sonchus bulbosus *(L.) Kilian & Greuter	spring	fields, uncultivated areas
*Sonchus oleraceus* (L.) L.	spring, winter	fields, pastures, roadsides
*Sonchus tenerrimus* L.	spring	fields, pastures, uncultivated areas
*Taraxacum campylodes* G.E. Haglund	autumn, spring, winter	pastures, rocky slopes
*Taraxacum minimum* (Briganti ex Guss.) N. Terracc.	autumn, spring, winter	pastures, rocky slopes
*Teucrium fruticans* L.	spring	escarpments, fields, rocky slopes
*Thymbra capitata* (L.) Cav.	spring, summer	escarpments, rocky slopes, uncultivated areas
*Tragopogon porrifolius* L.	spring	grassland, roadsides
*Trifolium phleoides* Willd.	spring	maquis, meadows, uncultivated areas
*Urospermum picroides* (L.) F.W. Schmidt	winter	roadsides, uncultivated areas
*Urtica dioica* L.	autumn, spring, winter	cultivated areas, roadsides
*Urtica membranacea* Poir. ex Savigny	winter	fields, roadsides, uncultivated areas
^a^ *Urtica rupestris* Guss.	autumn, spring, winter	cultivated areas, roadsides
*Urtica urens* L.	autumn, spring, summer	fields, roadsides
*Verbascum sinuatum* L.	spring	roadsides, uncultivated areas
*Wisteria sinensis* (Sims) Sweet	spring	roadsides, uncultivated areas

^a^endemism

### Most commonly cited wild food plant species

The Cultural Importance Index of the 119 species in the study varied between 0.004 and 0.50 (average value: 0.08) (Appendix).

With regards to the wild food plant species used in each of the Sicilian Regional Parks, the analysis shows that *Cichorium intybus* L. (C.I. 0.37), *Foeniculum vulgare* Miller (C.I. 0.31), *Borago officinalis* L. (C.I. 0.23) and *Asparagus acutifolius* L. (C.I. 0.21) are the most important species to the local populations in terms of food use (Table 2).

**Table 2:** Top 10 wild plant species most frequently cited for food purposes and currently used by informants in each of the four Sicilian Regional Parks, shown here the Cultural Importance Index

Plant species	Botanical family	Number of interview in which it was cited	Frequency (%)^a^	Cultural Importance Index
*Cichorium intybus* L.	Asteraceae	221	27.55	0.37
*Foeniculum vulgare* Mill.	Apiaceae	203	25.31	0.31
*Borago officinalis* L.	Boraginacae	149	18.57	0.23
*Asparagus acutifolius* L.	Asparagaceae	151	18.82	0.21
*Opuntia ficus-indica* (L.) Miller.	Cactaceae	143	18.00	0.19
*Sonchus oleraceus* (L.) L.	Asteraceae	144	17.95	0.19
*Clinopodium nepeta* (L.) Kuntze	Lamiaceae	127	15.83	0.16
*Ruscus aculeatus* L.	Asparagaceae	79	9.85	0.12
*Centaurea solstitialis* L. subsp. *schouwii* (DC.) Gugler	Asteraceae	75	9.35	0.11
*Laurus nobilis* L.	Lauraceae	75	9.35	0.09

^a^as a percentage of citations of the total of 802 informants

The results clearly demonstrate that the most frequently-cited species were also those most commonly-used for food purposes by the local people of the Parks involved.

*Cichorium intybus* was most cited by the people interviewed. The aerial parts and leaves of the species are eaten boiled and the broth is consumed as a drink. The boiled parts are used to prepare salads and soups in other regions of Italy such as Latium [71], Tuscany [72] and Sardinia [73]. In Sicily, the aerial parts of common chicory are also sautéed with eggs and seasoned with olive oil [14]. In the Madonie and Nebrodi Regional Parks in particular, it is widely used for pasta sauces. The aerial parts of *Foeniculum vulgare* are eaten boiled, seasoned with olive oil and lemon or consumed raw in salad. This food use is also confirmed by various regions in Italy [8, 12, 74] and in several countries in the Mediterranean as reported by [14]. The aerial parts of the species are also an ingredient in a number of traditional Sicilian pasta or meat dishes and soups. The aerial parts of *Borago officinalis* are eaten boiled and seasoned with olive oil and lemon, or fried in batter by local populations in the 4 Sicilian Parks. The boiled water, seasoned with olive oil, has diuretic and laxative properties, as reported in most of the literature from Italian regions and other Mediterranean countries [75–81]. The young turions of *Asparagus acutifolius* are eaten boiled and the boiled water consumed seasoned with olive oil. The turions may be fried with onions in omelettes, a dish particularly widespread in the Nebrodi and Monti Sicani Regional Parks but also common throughout Italy. The aerial parts of *Sonchus oleraceus* (L.) L. are eaten boiled and pan fried in omelettes. Lentini and Venza [14] reported that the young leaves are consumed in salads and soups in various Mediterranean counties such as Crete, Cyprus, Egypt, Spain and Tunisia. Of the more commonly cited species for each of the 4 Sicilian Parks, *Laurus nobilis* L. (C.I. 0.09) was found to have the lowest Cultural Importance Index. Most of the species had a very low Cultural Importance Index, this would seem to demonstrate that little cultural importance is given to these species as a food. This may be an indication of a fall in TPK (traditional plant knowledge) regarding food uses of plants. However, it would be a mistake to consider those species with a low C.I. index as uninteresting from a culinary point of view in that we would need to take other factors into consideration, such as the lesser or greater availability of the species in the natural habitat, or the greater or lesser use of the plants to cure the most widespread disorders in that same area. When considering the 90 towns in the study area individually, the cultural importance of these species was found to differ between towns, and this highlights the fact that, in quantitative analysis terms, the species is used by the local populations to differing degrees. In this study a total of 119 wild plant species were collected, 109 of which were found to be used for food purposes also in other Italian regions and 75 in the Mediterranean countries taken into consideration. Most previous studies, however, were carried out using different methodologies or in areas which differed in terms of size and floral diversity. When a comparison was made of the wild plant species in this study and those previously cited by Lentini and Venza [14] in Sicily, 65 were found to have been previously cited, whereas 54 appear to be newly mentioned. This is undoubtedly an important result for our study. When comparing the four Regional Parks, only 15 wild plant species were common to all of the parks. Most of the wild food plants were found in the Etna (65) and Nebrodi (62) Regional Parks whilst the fewest in the Sicani Regional Park (39). In general, our research indicates that 6 wild plant species have not been mentioned before in the Mediterranean area for culinary use. With reference to the culinary uses of wild plants, it was found that most of the culinary uses recorded were the same or similar to those in other parts of Sicily, as reported by [14], in other regions of Italy and some Mediterranean countries: *Apium nodiflorum* (L.) Lag., for example, is eaten in Sicily, Tuscany, Spain and Tunisia. It is worth noting that some culinary uses are typically found in only one Sicilian Park or another, and this would seem to be proof of a slight variation in diet between populations of the same region. The greatest range of culinary uses was found in the Etna (28) and Nebrodi Regional Parks (27). Regarding current use of the species cited in the study, 28.57 % of the species are still used today whilst the remaining part can be considered to have fallen out of use. Concerning the perceived degree of usefulness of the species for food purposes, and referring only to those species currently used by the local populations, Concerning the perceived degree of usefulness of the species for food purposes, and referring to those species currently used by the local populations, 59.66 % of the culinary uses were highly appreciated by the informants whilst only 54.62 % of the culinary uses were little appreciated. Some wild plants were recorded by the informants as being both highly and little appreciated.

### Plant parts used and methods of consumption

**Fig. 2:**
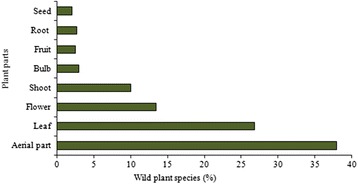
Frequency of plant parts used for food uses

**Fig. 3:**
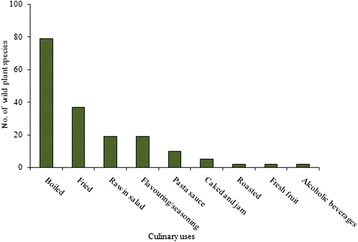
Main methods of consumption of the wild food plant species in the study area

The aerial parts of the plants are the parts most widely used, followed by leaves, flowers and shoots (Fig. 2); international literature also reports that the aerial parts and leaves are the most commonly used parts for culinary purpose, [14, 80, 81]. Greater accessibility in natural ecosystems of the aboveground parts of the plants and the greater abundance of the leaves compared to other plant parts may explain the higher use-frequency of these plants parts. In most cases, the various parts were used indifferently for the same culinary use only. For example, the leaves and the young shoots of *Silene vulgaris* (Moench) Garcke were used in quite the same way: eaten raw in salads or cooked in omelettes, and the aerials parts and flowers of *Asphodeline lutea* (L.) Rchb. were eaten indifferently, boiled and then fried with eggs. Further on the topic of preparation methods, the wild food species are consumed in a number of different ways: some are cooked whilst others are eaten raw and require only washing. The methods of consumptions are reported in Fig. 3. Most wild plant species are consumed boiled and nearly all (98.7 %) are eaten simply boiled and on their own. The most cited wild plant species eaten boiled are *Foeniculum vulgare*, *Borago officinalis* and *Asparagus acutifolius*. Some wild plant species are consumed fried, especially in the preparation of omelettes. In the Monti Sicani Regional Park, the aerial parts of *Diplotaxis erucoides* (L.) Dc. are typically fried with eggs and lemon in omelettes, and in the Nebrodi Regional Park the bulbs of *Leopoldia comosa* (L.) Parl. are sautéed with eggs to prepare traditional dishes as confirmed by [14]. A high number of plant species are consumed raw, most in salads. The aerial parts of *Portulaca oleracea* L., the young shoots of *Clematis vitalba* L., the leaves of *Nasturtium officinale* R. Br. are usually served with a little olive oil, salt and vinegar. Other species like *Allium* spp. and *Foeniculum vulgare* are used fresh with tomato and bread. Some wild plant species are consumed fried, especially in the preparation of omelettes. In the Monti Sicani Regional Park, the aerial parts of *Diplotaxis erucoides* (L.) Dc. are typically fried with eggs and lemon in omelettes, and in the Nebrodi Regional Park the bulbs of *Leopoldia comosa* (L.) Parl. are sautéed with eggs to prepare traditional dishes as confirmed by [14]. A high number of plant species are consumed raw, most in salads. The aerial parts of *Portulaca oleracea* L., the young shoots of *Clematis vitalba* L., the leaves of *Nasturtium officinale* R. Br. are usually served with a little olive oil, salt and vinegar. Other species like *Allium* spp. and *Foeniculum vulgare* are used fresh with tomato and bread. Some plants are consumed as fresh fruit: *Fragaria vesca* L. in Etna Regional Park and *Opuntia ficus-indica* (L.) Miller in the Madonie Regional Park. Fruits are also used to make cakes and preserves. Twelve plant species are used to prepare sauces for traditional Sicilian pasta recipes. The most representative are *Foeniculum vulgar*e, *Asparagus* spp., *Sonchu*s spp., *Borago officinalis* and *Capparis spinosa* L. Some aromatic plants are gathered in the wild and consumed steadily throughout during the year. The highly aromatic species *Origanum vulgare* L., *Rosmarinus officinalis* and *Thymbra capitata* (L.) Cav. are examples of such, commonly used to flavour traditional Sicilian dishes. Only two plants species are used for alcoholic beverages: in the Etna and Nebrodi Regional Parks, *Mentha pulegium* L. and *Mentha spicata* L. are traditionally used to make liqueurs.

### Food/medicinal wild plant species

Of the 119 wild plant species in this study, 64 were recorded as having therapeutic effects (Appendix). According to the informants, the dishes prepared and aromatized with these plants have additional health properties, in addition to that of nutrition. When considering 188 wild plant species used in traditional Sicilian cuisine, Lentini and Venza [14] noted that the majority were also used for medicinal purposes by the local populations. Similarities were found between their findings and the results of our study concerning plant species with food/medicinal properties, methods of consumption and medical-use preparation methods. Traditionally, food is often considered to be medicinal in Sicily, as remarked upon by [14], and the food use of a given species may also be seen as curative, depending on the gravity of the disorder. Guarrera and Savo [82] remark that wild food plants are often consumed for their health-giving or medicinal properties also in other parts of Italy. Furthermore, this strong relationship between food and medicinal uses according to [81] and [83], could help in developing of foods for functional, nutraceutical and medicinal purposes.

*Clinopodium nepeta* (L.) Kuntze was the species most cited by the populations of both the Etna and Nebrodi Regional Parks. The broth obtained from the aerial parts of the species is used to cure internal haemorrhoids. Singh et al. [84] note that the essential oil of *Clinopodium nepeta* contains 64 pharmacologically active compounds which give the species antibacterial, antioxidant and anti-inflammatory properties.

*Cichorium intybus* was the most-cited species by the populations of the Madonie and Monti Sicani Regional Parks. The aerial parts of this plant species are commonly boiled and eaten for their good flavour, but also as a bland diuretic, particularly highly appreciated by the local people. The same use was reported by [14]. The plant contains several active principles and is generally retained depurative and healthy as many other plants with a bitter taste. *Cichorium intybus* is well known in other Italian regions [12, 40, 76, 82], but also in Morocco [85], in Tunisia [78] and in Spain [70].

Several aromatic plants belonging to the Lamiaceae family were particularly highly-appreciated for their therapeutic effects by the informants. *Origanum vulgare* is an example of such; used in many traditional Sicilian recipes as flavouring in salads, or roast/fried fish and meats, it is also considered to be an important medicinal plant by the local populations, helping to fight colds, flu and stomachache [14]. Several studies carried out in Sicily on wild oregano plants in fact confirmed the antioxidant effect of the essential oils [86–88]. Other aromatic species, noted for their culinary and therapeutic uses, include *Rosmarinus officinalis* and *Thymbra capitata*; the leaves of both of these species are used to flavor vegetables, roast meats *etc.* A decoction of the aerial parts of rosemary is considered to be particularly effective in the treatment of asthma and gastric disorders; an observation commonly made in both Italian and world literature [89–92]. An infusion of thyme leaves is thought effective against coughs and colds, and in the treatment of gastrointestinal disorders. Research has found, however, that the quality and quantity of essential oil (which is responsible for the antioxidant and antimicrobial activity of the species) contained in wild rosemary and thyme, can vary considerably and appears to be significantly affected by both intrinsic and extrinsic factors to the species [93–95].

The culinary use of *Laurus nobilis* is extremely common throughout the study area. The fresh and dried leaves of the species are used for their distinctive flavor and fragrance. However, the species is also considered medicinal: laurel leaf infusions are used to help digestion, as noted in literature from Sicily [14], Italian regions and other Mediterranean countries [66, 91, 96]. Speroni et al. [97], in a study carried out on the gastro-protective effects and antioxidant properties of leaf extracts of laurel*,* noted that there seems to be a relationship between the pharmacological effectiveness of these species and its antiradical activity.

### Agriculture and wild food plant species

The local populations have very good knowledge of the cultivation techniques of small number of wild plant species and have wide experience with many agronomic practices such as soil preparation, crop rotation, sowing, fertilization, irrigation, diseases and pests and harvesting. Organic farming practices are also highy appreciated by the informants. In many towns of the Madonie Regional Park, common chicory and starflower are commonly grown in kitchen gardens and the most used cultivation techniques for these crops are similar to that of spinach and salad rocket. The increasing importance of fennel in the Mediterranean diet has encouraged many aromatic plant producers to breed and commercialize the species in pots. The cultivation of cactus pear is especially widespread in the Etna Regional Park and, on a European level, cactus pear processing is only found in Sicily. The success of this Sicilian production sector is the result of excellent quality fruit, but also in part due to the recent adoption of low-impact cultivation techniques, such as fertilisation, irrigation and thinning, which traditionally were not practiced on this species. In recent years, in the most productive agricultural areas of Sicily, cactus pear crops have been cultivated with irrigation, greatly improving fruit quality.

Laurel is grown in kitchen gardens and farms for the production of aromatic plants. Regarding all the 119 wild plant species, it is important to note that the majority are not cultivated and only a small number are cultivated or could be cultivated in kitchen gardens and crop fields. Another point worth highlighting is the fact that agronomic practices often affect organoleptic and nutritional properties of the plant parts. This may depend on how extensive cultivation is: the more intensive production, the greater the probability of a change occurring in the nutritional properties of the food. Many informants commented on the fact that *wild* plant species were beneficial to human health because they were not contaminated by fertilizers and pesticides, and that they contained large amounts of nutrients and active principles. Vice versa informants stated that the domesticated plants were not grown naturally and could possibly contain substances harmful to health as a result of human intervention. Most of the informants perceived that plants cultivated in own-kitchen gardens were better than those produced in intensive farming. However, from an agronomic point of view, the domestication of wild plant species, using low impact cultivation techniques, represents a point of interest for sustainable agriculture in order to obtain a high quality foods for human consumption. Nowadays, many consumers want to know more about the food they eat and look for high quality. Agriculture needs to ensure organoleptic stability of a wild plant species when cultivated on a large scale. The first step is to protect the plant genetic resources through ex situ conservation, and the second is to establish cultivation protocols for the wild plant species. In this way, it is possible to select wild plant species with agronomic interest for food/medicinal uses.

## Conclusions

This study carried out in 4 Regional Parks in Sicily shows that the culinary use of wild shrub and herbaceous plants is not a predominant part of the food culture of the local populations. Most of the species were not found in all of the Sicilian Parks and the number of total citations per species was, in general, very low: sign of an on-going process of the cultural erosion. The lack of homogeneity in the number of women and men in the interview group may have affected some results of the study; in particular, the under-representation of women in the sample of informants is most likely to have contributed to lower quantities of detailed information, for example on the methods of consumption of wild plants. The study found that only very few wild plants are widely used for food use by the elderly populations of the study area and, on many occasions, their consumption is due to the fact that wild plants are used both for food and medicinal purposes. This confirms the fact that, in Sicily, some foods are believed to have a natural, therapeutic effect and the food use of one species or another is considered curative of various disorders. Quantitative analysis shows that the plants that have the greatest probability of survival over time *as a source of food* are those at the interface of food and medicine, and not those used solely as a food. Comparative analysis with other Mediterranean regions indicates that 6 wild food species have not been mentioned previously in the literature of the references countries and would seem to be novel in culinary terms. In terms of agriculture, it is important to highlight that given the fact that only very few of the wild plants mentioned can/could be cultivated in kitchen gardens and/or crop fields, further agronomic research on these few species is essential in order to improve knowledge on their main cultivation techniques. An important result of the research is the fact that most of the wild plants are perceived as highly useful for food/medicinal purposes and this is due to the health effects of the wild plants as reported by the informants. The protection of the native genetic resources and the culinary traditions linked to them is essential if we are to preserve the cultural heritage of the Sicilian Parks concerning the food use of wild plant species and in order to cultivate a number of species of agricultural interest. Our contribution should be not considered as exhaustive and future research is necessary in order to extend investigation to the younger generations and comment on the transmission of knowledge from the old to the new generation.

## Appendix

**Table 3:** List of the wild plant species used for food purposes in the study area

Family, scientific name, voucher specimen code	Vernacular name (in Sicilian dialect)	Part(s) of plant used	Food uses in the study area	Presence of the species in the study area	Use reports	Current use in the study area	Cultural importance index	Level of perceived usefulness of species	Species recorded for food purpose also in other Italian regions	Species recorded for food purposes also in other Mediterranean countries	Species previously cited for food purposes in Sicily	Medicinal uses in the study area
Amaryllidaceae												
*Allium ampeloprasum L. (CORISSIA - S/0392)*	porracciu	Bulb	Flavouring, raw in salad	M	20	P	0.13	H	X	Y	Z	
*Allium subhirsutum L. (CORISSIA - S/0017)*	agghiu sarvaggiu	Aerial part, bulb	Flavouring, raw in salad	N	14	P	0.06	H	X			
Apiaceae												
*Anthriscus nemorosa (M. Bieb.) Spreng. (CORISSIA - S/0089)*	coscia di cunigghiu	Aerial part	Boiled vegetables	E	1		0.005	L	X			
*Ammi majus L. (CORISSIA - S/0204)*	ennera	Aerial part	Boiled vegetables	N	1		0.004	H	X	Y		
*Apium nodiflorum (L.) Lag. (CORISSIA - S/0090)*	scavuni, sedanu	Aerial part	Boiled vegetables	M	24	P	0.16	H	X	Y	Z	Diuretic (M)
*Foeniculum vulgare Miller (CORISSIA - S/0038)*	finucchieddu, finucchieddu rizzu	Aerial part, seed	Boiled vegetables, raw in salad, sauce for pasta	E, M, N, S	248	P	0.31	H	X	Y	Z	Digestive, gastrointestinal disorders (E, M, N, S)
*Oenanthe pimpinelloides L. (CORISSIA - S/0293)*	finocchiu acquaticu	Aerial part	Boiled vegetables	M	5		0.03	H	X			
Aristolochiaceae												
*Aristolochia sempervirens L. (CORISSIA - S/0230)*	cannatedda	Leaf	Boiled vegetables	E	50		0.25	H		Y		
Asparagaceae												
*Asparagus albus L. (CORISSIA - S/0228)*	sparacio bianco, sparaciu biancu spinosu	Turion	Boiled vegetables, omelettes, sauce for pasta	E, M, S	54	P	0.09	H, L	X	Y	Z	Diuretic (E, M, S)
*Asparagus aphillus L. (CORISSIA - S/0043)*	aparaggiu servaggiu, sparaci di bruscu	Aerial part, turion	Boiled vegetables	E, N	8		0.02	H	X	Y		
*Asparagus acutifolius L. (CORISSIA - S/0202)*	sparaciu nivuru, sparaciu servaggiu	Turion	Boiled vegetables, omelettes, sauce for pasta	E, M, N, S	167	P	0.21	H	X	Y	Z	Diuretic (E, M, N, S)
*Asparagus horridus L. (CORISSIA -S/0249)*	sparacogni	Turion	Boiled vegetables, fried as vegetables with eggs	N	32	P	0.14	H	X			
*Leopoldia comosa (L.) Parl. (CORISSIA - S/0368)*	cipuddazzu, giacintu sarvaggiu	Aerial part, bulb	Boiled bulbs, fried bulbs with eggs, onion balls	N	8		0.03	L	X	Y	Z	
*Muscari botryoides (L.) Mill. (CORISSIA - S/0385)*	capudazza	Bulb	Flavouring, seasoning	E	2		0.01	L	X			
*Polygonatum multiflorum (L.) All. (CORISSIA - S/0393)*	sparaci i curma	Aerial part	Boiled vegetables, fried as vegetables with eggs	N	3		0.01	L				
*Ruscus aculeatus L. (CORISSIA - S/0363)*	asparagi, spinapulici, pungitopo, sparaciu di tronu	Turion	Boiled vegetables, fried as vegetables with eggs	E, M, N, S	97	P	0.12	H, L	X	Y	Z	Diuretic (E, S)
Asteraceae												
*Artemisia alba Turra (CORISSIA - S/0215)*	canforedda	Leaf, flower	Flavouring	E	1		0.005	L	X			Mineralizing (E)
*Calendula arvensis (Vaill.) L. (CORISSIA - S/0184)*	calennula	Flower	Boiled vegetables	N	1		0.004	L	X	Y		
*Carduus argyroa Biv. (CORISSIA - S/0189)*	napodi d'acqua, napordio	Flower	Boiled vegetables, fried as vegetables with eggs	S	9		0.04	H	X	Y	Z	
*Carduus corymbosus Ten. (CORISSIA - S/0394)*	carvi	Flower, seed	Omelettes	E	1		0.005	H	X			
*Carlina corymbosa L. (CORISSIA - S/0395)*	mazzacani	Flower	Boiled vegetables	E	2		0.01	L	X			
*Carlina gummifera L. (Less.) (CORISSIA - S/0316)*	masticogna	Inflorescence, root	Boiled vegetables, raw in salad	M, N, S	22		0.04	H, L	X			
*Carlina sicula Ten. (CORISSIA -S/0284)*	pani cauru	Aerial part	Boiled vegetables, fried as vegetables with eggs	M, N	17		0.04	H, L			Z	
*Carthamus pinnatus Desf. (CORISSIA - S/0396)*	cardunceddu	Aerial part	Boiled and fried vegetables, raw in salad	M	3		0.02	L	X			
*Centaurea solstitialis L. subsp. schouwii (DC.) Gugler (CORISSIA - S/0078)*	aprocchio, apuarchiu, procchia	Leaf, whole plant	Boiled vegetables, fried as vegetables with eggs	E, M, N, S	85	P	0.11	H, L	X	Y	Z	Diuretic (M), stomach pains (N)
*Chondrilla juncea L. (CORISSIA - S/0389)*	cutulidda	Aerial part	Boiled vegetables	E	5		0.02	H	X	Y	Z	
*Cichorium intybus L. (CORISSIA - S/0026)*	ciconia, cicoria, cicuaria	Aerial part, leaf	Boiled vegetables, fried as vegetables with eggs, sauce for pasta	E, M, N, S	295	P	0.37	H	X	Y	Z	Diuretic (E, M, N, S), laxative (M, N)
*Crepis vesicaria L. (CORISSIA - S/0036)*	cicuriuni	Aerial part	Boiled vegetables	S	7		0.03	L	X	Y	Z	
*Helianthus tuberosus L. (CORISSIA - S/0240)*	cazzatummula	Tuber	Cooked with sweet potatoes	N	3		0.01	L	X	Y		
*Helminthotheca echioides (L.) Holub (CORISSIA - S/0397)*	aspragini vulgari	Aerial part	Boiled vegetables, omelettes, sauce for pasta	M	7		0.05	L				
*Hyoseris radiata L. (CORISSIA - S/0070)*	cicoria sarvaggia	Aerial part	Boiled vegetables	S	3		0.01	L	X		Z	Diuretic (S)
*Hypochoeris radicata L. (CORISSIA - S/0283)*	coscivecchi, costi i vecchia, costolina	Inflorescence, leaf	Boiled vegetables	E, M, N	103	P	0.18	H, L	X	Y	Z	Kidney stones, renal colics (E)
*Lactuca viminea (L.) J. & C. Presl (CORISSIA - S/0290)*	cacciacunigghiu, pedi di nigghiu	Aerial part, leaf	Raw in salad	E, N	8		0.02	H	X	Y	Z	
*Notobasis syriaca (L.) Cass. (CORISSIA - S/0007)*	piscialasinu, spina ianca	Inflorescence	Boiled and gratin vegetables, fried as vegetables with eggs, omelettes	N, S	8		0.01	L	X		Z	
*Onopordum illyricum L. (CORISSIA -S/0197)*	minapurdi,munaceddi, napordi, onopordo	Aerial part, leaf, stem	Boiled vegetables, dipped in flour and eggs	M, N, S	95		0.16	H	X	Y	Z	
*Reichardia picroides (L.) Roth (CORISSIA - S/0192)*	caccialebra, grattalingua comune	Aerial part	Boiled vegetables	E, M	50	P	0.14	H	X	Y	Z	Headache (E)
*Scolymus grandiflorus Desf. (CORISSIA - S/0160)*	cardagna maggiore	Aerial part, stem	Boiled vegetables, raw in salad	E, M	51		0.14	H	X		Z	
*Scolymus hispanicus L. (CORISSIA - S/0398)*	scoddi	Aerial part	Boiled vegetables	N	3		0.01	L	X	Y	Z	
*Scolymus maculatus L. (CORISSIA - S/0399)*	cacalasagni	Stem	Raw in salad, omelettes,sauce for pasta	S	16		0.07	H, L	X	Y	Z	
*Scorzonera hispanica L. (CORISSIA - S/0049)*	scussunara	Aerial part	Boiled vegetables	E	12		0.06	H		Y		
*Silybum marianum (L.) Gaertner (CORISSIA - S/0212)*	buanazzi, cardu	Aerial part	Boiled vegetables, raw in salad	M, N	17		0.04	L	X	Y	Z	Liver disorders, stomach pains (N)
*Sonchus asper (L.) Hill (CORISSIA - S/0077)*	cardedda spinosa, cardedda sarvaggia, crispinu spinosu	Aerial part, leaf	Boiled vegetables, sauce for pasta	E, M, S	58		0.10	H	X	Y	Z	Digestive (S)
*Sonchus bulbosus (L.) Kilian & Greuter (CORISSIA - S/0400)*	latte d'aceddu	Leaf	Mixed vegetables	E	1		0.01	L	X			
*Sonchus oleraceus (L.) L. (CORISSIA - S/0364)*	cardera duci, cardedda, crespino comune	Aerial part, whole plant	Boiled vegetables, flavouring, omelettes, sauce for pasta	E, M, N, S	154	P	0.19	H, L	X	Y	Z	Digestive, refreshing (M, N, S)
*Sonchus tenerrimus L. (CORISSIA - S/0010)*	crespino	Aerial part	Boiled vegetables, sauce for pasta	M	9		0.06	L	X	Y	Z	
*Taraxacum campylodes G.E. Haglund (CORISSIA - S/0032)*	denti di liuni	Inflorescence	Boiled vegetables, sautéed vegetables wit batter	N	13		0.05	H, L	X	Y		
*Taraxacum minimum (Briganti ex Guss.) N. Terracc. (CORISSIA - S/0069)*	cicoria sarvaggia, pedi d'aceddu	Inflorescence	Boiled vegetables, raw in salad	N	3		0.01	L	X			
*Tragopogon porrifolius L. (CORISSIA -S/0052)*	nzitarola, perciacannedda	Inflorescence, leaf, root	Boiled vegetables, raw in salad	N	3		0.01	L	X	Y	Z	
*Urospermum picroides (L.) F. W. Schmidt (CORISSIA - S/0087)*	coccialebbra, coccidilepre	Leaf	Boiled vegetables	S	2		0.008	L	X	Y	Z	
Boraginaceae												
*Borago officinalis L. (CORISSIA - S/0268)*	burrania, borragine, vurrania	Aerial part	Boiled vegetables, sauce for pasta, sautéed vegetables with batter	E, M, N, S	185		0.23	H	X	Y	Z	Diuretic, laxative (E, M, N, S), digestive (N)
*Echium vulgare L. (CORISSIA - S/0106)*	viperina	Aerial part, inflorescence	Boiled vegetables	N	1		0	L	X	Y		
*Heliotropium europaeum L. (CORISSIA - S/0313)*	erba i nasu	Aerial part	Boiled vegetables	E	1		0.01	L				
Brassicaceae												
*Arabis hirsuta (L.) Scop. (CORISSIA - S/0051)*	razzi	Aerial part	Boiled vegetables	E	29		0.15	H	X			
*Barbarea vulgaris R. Br. (CORISSIA - S/0220)*	mazzarelli	Young shoots	Boiled vegetables	E	2		0.01	L	X			
*Brassica fruticulosa Cirillo (CORISSIA - S/0093)*	caliceddi, caluceddi, calicelli	Aerial part, leaf	Boiled vegetables, sautéed vegetables with batter	E	98	P	0.50	H	X	Y	Z	
*Brassica rupestris Raf. (CORISSIA - S/0101)*	caliceddi i muru, cavulazzu	Leaf	Boiled vegetables, fried as vegetables with eggs	E, N	8		0.02	H, L	X			
*Bunias erucago L. (CORISSIA - S/0381)*	cicoria selvaggia	Aerial part	Boiled vegetables	E	62		0.32	H	X		Z	Digestive, diuretic (E)
*Diplotaxis erucoides (L.) Dc. (CORISSIA - S/0286)*	arsaneddi d'aglia, caluzzi di vigna, lassaneddi d'aglia, lassanu d'aglia, pissineddi, sinacciola	Aerial part	Boiled vegetables, fried as vegetables with eggs and lemons	S	37		0.16	H	X	Y		
*Diplotaxis harra (Forssk.) Boiss. (CORISSIA - S/0345)*	cavuliceddi	Leaf	Boiled vegetables	S	3		0.01	L		Y	Z	
*Isatis tinctoria L. (CORISSIA - S/0372)*	cavolu carrammu	Flower	Boiled vegetables	E	2		0.01	L	X			
*Nasturtium officinale R. Br. (CORISSIA - S/0140)*	crescione, crisciuni,	Aerial part, leaf	Boiled vegetables, raw in salad	E, M, N, S	45	P	0.05	H, L	X	Y	Z	Diuretic (E, S, N), renal pains (N)
*Raphanus raphanistrum L. (CORISSIA - S/0258)*	mazzaredda, ravaneddu, razzi	Aerial part	Boiled vegetables	M, N, S	33		0.05	H	X	Y	Z	
*Sinapis alba L. (CORISSIA - S/0168)*	azzareddi, mazzarello bianco	Aerial part	Boiled vegetables	E, S	20	P	0.04	H	X	Y	Z	
*Sinapis arvensis L. (CORISSIA - S/0057)*	cavulazzu, pisciacunigghiu	Aerial part, seed	Boiled vegetables, sautéed vegetables with batter	E, M, N, S	64	P	0.08	H	X	Y	Z	Bronchitis, cough (N)
*Sinapis pubescens L. (CORISSIA - S/0042)*	sinapi	Aerial part	Sautéed vegetables with batter	M	5		0.03	H	X			
*Sisymbrium irio L. (CORISSIA - S/0375)*	pisciacani, assini	Aerial part	Boiled vegetables, fried as vegetables with eggs	N	13		0.05	L	X	Y	Z	
Cactaceae												
*Opuntia ficus-indica (L.) Miller (CORISSIA -S/0046)*	ficu d'innia, ficudinia	Fruit	Fresh fruit, marmalade	E, M, N, S	157	P	0.19	H	X	Y	Z	Digestive (E, M, N, S), diuretic (E, M, N, S)
Capparaceae												
*Capparis spinosa L. (CORISSIA - S/0037)*	chiapparu, chiapperi, chiapparedda	Flower bud	Raw in salad, sauce for pasta, fish and meet	E, M, N, S	33	P	0.04	H	X	Y	Z	
Caprifoliaceae												
*Fedia cornucopiae (L.) Gaertn. (CORISSIA - S/0014)*	spezzacannati	Aerial part, young shoot	Raw in salad	M, N	10		0.03	L	X		Z	
*Scabiosa columbaria L. (CORISSIA - S/0044)*	erva di cavaleri	Leaf	Boiled vegetables	E	1		0.01	L	X			
Caryophillaceae												
*Silene vulgaris (Moench) Garcke (CORISSIA - S/0080)*	aricchi i lepri, erva privicatura	Leaf, young shoot	Omelettes, raw in salad	E, N	27		0.06	H	X	Y	Z	
Convolvulaceae												
*Calystegia sepium (L.) R. Br. (CORISSIA - S/0401)*	campaneddu	Leaf	Boiled vegetables	E	10		0.05	H	X			
Dioscoreaceae												
*Dioscorea communis L. Caddick & Wilkin (CORISSIA - S/0003)*	sparaci i curriola, sparaci ri serpa, viddicedda, vitarra	Aerial part, young shoot	Fried as vegetables with eggs	M, N, S	52		0.08	H	X			Digestive (N, S), laxative (M, N),
Equisetaceae												
*Equisetum arvense L. (CORISSIA - S/0349)*	cuda i cavaddu	Aerial part, leaf	Boiled vegetables	E, N	6		0.01	H	X	Y		Diuretic (N)
Geraniaceae												
*Erodium moschatum (L.) L'Hér. (CORISSIA - S/0185)*	panizzi da bedda matri	Aerial part	Boiled vegetables	N	1		0.004	L	X	Y		
Iridaceae												
*Crocus biflorus Mill. (CORISSIA - S/0341)*	ciuri pi fari u zafferanu	Flower	Flavouring	E	1		0.01	L	X	Y		
*Gladiolus italicus Mill. (CORISSIA - S/0321)*	cutiddi	Aerial part	Boiled vegetables	E	1		0.01	L	X			
Lamiaceae												
*Clinopodium nepeta (L.) Kuntze (CORISSIA - S/0149)*	niputedda, nitedda, nepotella	Leaf	Flavouring	E, M, N, S	127	P	0.16	H, L	X	Y	Z	Heamorroids (E)
*Marrubium vulgare L. (CORISSIA - S/0307)*	marrobbiu	Leaf	Boiled vegetables	S	1		0	L	X	Y		Bronchitis, cough (S)
*Mentha pulegium L. (CORISSIA - S/0229)*	iuri di menta	Leaf	Flavouring, liqueurs, seasoning	E, N	17	P	0.02	H	X	Y	Z	Digestive (N)
*Mentha spicata L. (CORISSIA - 0198)*	menta	Leaf	Flavouring, liqueurs, seasoning	E	4	P	0.02	L	X	Y	Z	
*Nepeta cataria L. (CORISSIA - S/0041)*	citulella	Aerial part	Boiled vegetables	E	4		0.02	H	X			Sedative (E)
*Origanum vulgare L. (CORISSIA - S/0398)*	arianu, rianu	Flower, leaf	Flavouring, seasoning	E, N, S	110	P	0.17	H	X	Y	Z	Digestive (N)
*Phlomis fruticosa L. (CORISSIA - S/0287)*	sarvia sarvaggia	Leaf	Flavouring	S	2		0.01	L	X	Y	Z	
*Rosmarinus officinalis L. (CORISSIA - S/0357)*	rosmarinu	Aerial part, leaf	Flavouring, seasoning	E, N, S	109	P	0.16	H	X	Y	Z	Asthma (N, S), digestive, stomach pains (N)
*Teucrium fruticans L. (CORISSIA - S/0231)*	caccazzina, ricuttedda	Aerial part	Sautéed vegetables with eggs and batter	E, N	16		0.04	H	X	Y	Z	Heamorroids (E)
*Thymbra capitata (L.) Cav. (CORISSIA - S/0391)*	sataredda	Flower, leaf	Flavouring, seasoning	E, M, N, S	38	P	0.04	H	X	Y	Z	Sedative (S), throat and mouth inflammations (N)
Lauraceae												
*Laurus nobilis L. (CORISSIA - S/0218)*	dauru, davuru	Leaf	Flavouring	E, M, N, S	75	P	0.09	H	X	Y	Z	Airways inflammation, cold, cough (M, N), digestive (E, M, N, S)
Leguminosae												
*Anagyris foetida L. (CORISSIA - S/0068)*	fasolu taddi	Shoot	Fried as vegetables with eggs	N	6		0.03	L	X			
*Glycirrhiza glabra L. (CORISSIA - S/0354)*	niculizia	Root	Raw in salad	N, S	15	P	0.03	H	X	Y	Z	Cold, cough (N, S)
*Lathyrus clymenum L. (CORISSIA - S/0402)*	fasolu taddi	Shoot	Fried as vegetables with eggs	N	6		0.03	H	X	Y	Z	
*Lathyrus sativus L. (CORISSIA -S/0132)*	chiecchiera	Fruit	Raw in salad	N	2		0.009	H	X	Y		
*Trifolium phleoides Willd. (CORISSIA - S/0223)*	cuda di surciu, curi ri succi	Aerial part	Boiled vegetables, fried as vegetables with eggs	E, N	17		0.04	H, L	X	Y		Diuretic (E)
*Wisteria sinensis (Sims) Sweet (CORISSIA - S/0187)*	glicine	Aerial part , inflorescence	Sautéed vegetables with batter	N	2		0.009	L				
Malvaceae												
*Malva sylvestris L. (CORISSIA - S/0227)*	marva	Leaf	Boiled vegetables	E	16		0.08	H	X	Y	Z	Anti-inflammatory, digestive, diuretic, haemorroids, renal colics (E)
Oxalidaceae												
*Oxalis pes-caprae L. (CORISSIA - S/0244)*	acetosella, castanziculli, castagnola	Leaf, bulb, root	Side dish, roast like chestnuts	E, M, N	12		0.02	H, L	X	Y	Z	Digestive (N)
Orobanchaceae												
*Orobanche crenata Forssk (CORISSIA - S/0209)*	lupa	Aerial part, stem	Raw in salad	N, S	5		0.01	L	X			Cough (N)
Plantaginaceae												
*Globularia alypum L. (CORISSIA - S/0334)*	erva bianca	Leaf	Boiled vegetables	E	15		0.08	H	X			Renal stones (E)
*Plantago lagopus L. (CORISSIA - S/0165)*	coda di gatto	Aerial part	Boiled vegetables	E	16		0.08	H	X	Y	Z	
*Plantago coronopus L. (CORISSIA - S/0278)*	erva stidda	Aerial part, leaf	Boiled vegetables	N	2		0.009	L	X	Y		Astringent (N)
Poaceae												
*Elymus repens (L.) Gould. (CORISSIA - S/0028)*	gramigna	Whole plant	Boiled vegetables	E	51		0.26	H	X			Diuretic, hypertension, renal colics, stomach pains (E)
Polypodiaceae												
*Polypodium vulgare L. (CORISSIA - S/0327)*	filici	Leaf, root	Boiled vegetables	E	3		0.01	L	X			Depurative, digestive, laxative (E)
Portulacaceae												
*Portulaca oleracea L. (CORISSIA - S/0369)*	purciddana	Aerial part	Raw in salad	E, M, N, S	64	P	0.08	H, L	X	Y	Z	Depurative (E)
Ranunculaceae												
*Clematis cirrhosa L. (CORISSIA - S/0335)*	viterbi	Leaf	Boiled vegetables	E	1		0.01	L				
*Clematis vitalba L. (CORISSIA - S/0328)*	aliferi, clematidi	Turion	Mixed vegetables, omelettes, raw in salad	M	20		0.13	L	X	Y	Z	
Rosaceae												
*Fragaria vesca L. (CORISSIA - S/0362)*	fraula silvatica	Fruit, leaf	Fresh fruit, marmalade	E, M, N	23	P	0.04	H	X	Y		Diuretic (E)
*Rosa canina L. (CORISSIA - S/0061)*	rusera	Flower, fruit	Marmalade	M, N	17		0.04	L	X	Y	Z	Digestive (N)
*Rubus ulmifolius Schott (CORISSIA - S/0261)*	amuredda, rivetta, rovi, ruvetta	Fruit, flower, leaf	Cakes, marmalade	M, N, S	60	P	0.09	H, L	X	Y	Z	Haemorrhoids (S)
Rubiaceae												
*Asperula rupestris Tineo (CORISSIA - S/0193)*	spuredda	Leaf	Boiled vegetables	N	2		0.009	L				
Scrophulariaceae												
*Verbascum sinuatum L. (CORISSIA - S/0180)*	erba de morroidi	Leaf	Boiled vegetables	E	2		0.01	L	X			Haemorroids (E)
Smilacaceae												
*Smilax aspera L. (CORISSIA - S/0073)*	salsapariglia	Shoot	Boiled vegetables, sautéed vegetables with batter	M, N	40	P	0.11	H, L	X	Y	Z	
Solanaceae												
*Lycium europaeum L. (CORISSIA - S/0166)*	spina santa	Young shoot	Sautéed vegetables with batter	S	3		0.01	L	X	Y	Z	Digestive, refreshing (S)
Urticaceae												
*Parietaria officinalis L. (CORISSIA - S/0127)*	erva i ventu	Leaf	Boiled vegetables	E, N	4		0.009	H	X			Cough, diuretic, headache, hypertension, stomach pains (E)
*Urtica dioica L. (CORISSIA - S/0260)*	lardica, ortica	Leaf	Mixed vegetables, omelettes, raw in salad	M, N	76	P	0.20	H, L	X	Y		Anti-dandruff, anaemia (M), abscess, digestive (N)
*Urtica membranacea Poir. ex Savigny (CORISSIA - S/0392)*	ferdica	Aerial part	Raw in salad	S	1		0	L	X	Y	Z	Anti-inflammatory, digestive, refreshing (S)
*Urtica urens L. (CORISSIA - S/0017)*	ardicula	Aerial part	Boiled vegetables, mixed vegetables with legumes	S	18		0.08	L	X	Y	Z	Digestive, refreshing (S)
*Urtica rupestris Guss. (CORISSIA - S/0169)*	ardica	Leaf	Boiled vegetables	E	24	P	0.12	H	X			Digestive, haemorroids, renal colics (E)
Xanthorrhoeaceae												
*Asphodeline lutea (L.) Rchb. (CORISSIA - S/0259)*	asfodelo giallo, scannabecchi, scornabecchi	Aerial part, inflorescence	Boiled vegetables, fried as vegetables with eggs	E, M, N, S	55	P	0.07	H	X		Z	Diuretic (E)
*Asphodelus ramosus L. (CORISSIA - S/0403)*	erva di oliva	Leaf	Boiled vegetables	E	6		0.03	H	X		Z	

Abbreviations

Symbols: *H* high level of perceived usefulness, *L* low level of perceived usefulness, *P* current use in the study area, *X* food use recorded also in other Italian regions, *Y* food use recorded also in other Mediterranean countries, *Z* species previously cited for food purposes in Sicily

Sicilian Regional Parks: E - Etna Regional Park; M - Madonie Regional Park; N - Nebrodi Regional Park; S - Monti Sicani Regional Park

References for Italian regions:

Atzei (1991); Ilardi and Raimondo [37]; Pieroni [104]; Lentini [40]; Pieroni [8]; Pieroni [72]; Guarrera [71]; Atzei [73]; Pieroni et al. [9]; Guarrera and Manzi [74]; Guarrera [108]; Guarrera et al. [12]; Lentini and Venza [14]; Pieroni (2011); Guarrera and Savo [82]; Ranfa et al. [28]; Sansanelli and Tassoni [7]

References for Mediterranean countries:

Jouad et al. [85]; Bonet and Vallès [81]; Ertuğ [99]; Dogan et al. [98]; Rivera et al. [10]; Tardío et al. [67]; Della et al. [11]; Ozbucak et al. [102]; Pardo-de-Santayana et al. [103]; Pardo-de-Santayana et al. (2007); Ali-Shtayeh et al. [68]; Leporatti and Ghedira [78]; Menendez-Baceta et al. [100]; Nassif and Tanji [101]; Powell et al. [105]
